# Letter from the Editor in Chief

**DOI:** 10.19102/icrm.2022.130608

**Published:** 2022-06-15

**Authors:** Moussa Mansour


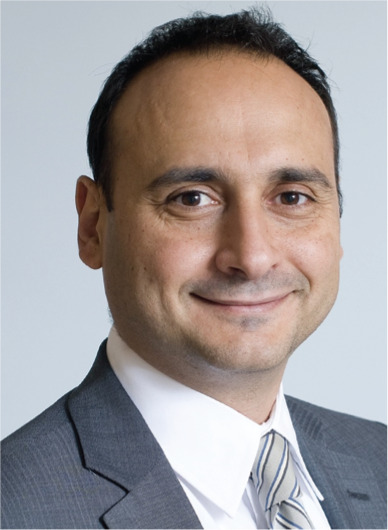


Dear readers,

In this issue of *The Journal of Innovations in Cardiac Rhythm Management*, I would like to highlight the paper by Drs. Gill and Wu titled “In-hospital Outcomes and Arrhythmia Burden in Patients with Obstructive Sleep Apnea and Heart Failure with Preserved Ejection Fraction.” This study investigated the outcomes and occurrence of arrhythmias in patients with heart failure and preserved ejection (HFpEF) fraction with and without sleep apnea. The authors searched a large national inpatient database to identify 127,773 hospitalizations of patients with HFpEF, 20% of whom had sleep apnea. It was concluded that patients with sleep apnea had a higher mortality rate, a longer duration of hospitalization, and greater medical costs. In addition, sleep apnea was associated with higher incidence rates of arrhythmias, including atrial fibrillation (AF). Sleep apnea treatment was not associated with improved outcome; however, the number of patients treated was relatively small. Although this was an observational retrospective study, limiting the impact of its findings, it highlights the influence of a modifiable risk factor, sleep apnea, on the incidence of cardiac arrhythmias, specifically AF.

Data have demonstrated that different risk factors, including obesity, sleep apnea, and alcohol consumption, are associated with an increased incidence of AF, and many randomized studies suggest that controlling these risk factors can reduce the burden of AF. For example, abstinence from alcohol reduced arrhythmia recurrences in regular drinkers.^1^ The outcome of catheter ablation for AF in patients with sleep apnea was also better in patients with continuous positive airway pressure therapy than those without.^2^ As a result, major societies have incorporated risk factor modification into their guidelines for the management of AF. For example, the European Society of Cardiology designed identification and management of risk factors as a class I indication for the management of AF.^3^

Despite the availability of high-quality data, however, risk factor modification for AF remains an elusive goal, and many catheter ablation procedures are performed without an attempt to control the above-mentioned risk factors. It is difficult to control AF risk factors because many involve life-long habits that are hard to change. Risk factor modification requires long-term interventions that are currently poorly incentivized and likely only to succeed if integrated into a comprehensive, multidisciplinary AF management plan.

I hope you enjoy reading this issue of *The Journal of Innovations in Cardiac Rhythm Management*, and I wish you a relaxing summer.


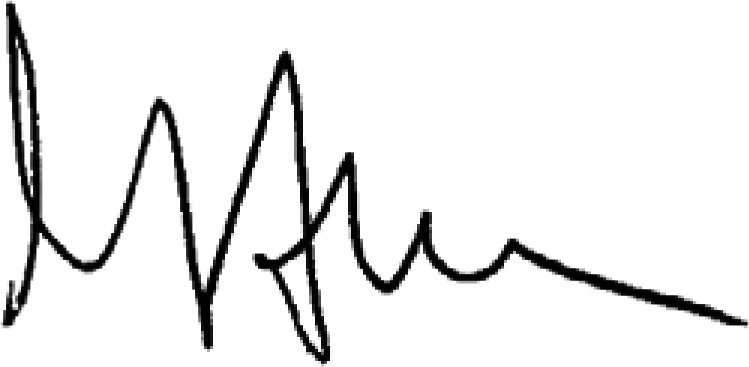


Sincerely,

Moussa Mansour, md, fhrs, facc

Editor in Chief

*The Journal of Innovations in Cardiac Rhythm Management*

MMansour@InnovationsInCRM.com

Director, Atrial Fibrillation Program

Jeremy Ruskin and Dan Starks Endowed Chair in Cardiology

Massachusetts General Hospital

Boston, MA 02114

## References

[1] Voskoboinik A, Kalman JM, De Silva A (2020). Alcohol abstinence in drinkers with atrial fibrillation. N Engl J Med.

[2] Fein AS, Shvilkin A, Shah D (2013). Treatment of obstructive sleep apnea reduces the risk of atrial fibrillation recurrence after catheter ablation. J Am Coll Cardiol.

[3] Hindricks G, Potpara T, Dagres N (2021). 2020 ESC Guidelines for the diagnosis and management of atrial fibrillation developed in collaboration with the European Association for Cardio-Thoracic Surgery (EACTS): The Task Force for the diagnosis and management of atrial fibrillation of the European Society of Cardiology (ESC) Developed with the special contribution of the European Heart Rhythm Association (EHRA) of the ESC. Eur Heart J.

